# Understanding noble gas incorporation in mantle minerals: an atomistic study

**DOI:** 10.1038/s41598-024-61963-x

**Published:** 2024-06-12

**Authors:** Alfredo Lora, Paola Patron, Alin M. Elena, Neil L. Allan, Carlos Pinilla

**Affiliations:** 1 Departamento de Fisica y Geociencias, Universidad del Norte, km 5 Via Puerto Colombia, Barranquilla, Colombia; 2 Computational Chemistry Group, STFC Daresbury Laboratory, Keckwick Lane, Daresbury, WA4 4AD UK; 3 School of Chemistry, University of Bristol, Cantock’s Close, Bristol, BS8 1TS UK

**Keywords:** Geochemistry, Mineralogy, Computational methods, Atomistic models

## Abstract

Ab initio calculations in forsterite (Mg$_2$SiO$_4$) are used to gain insight into the formation of point defects and incorporation of noble gases. We calculate the enthalpies of incorporation both at pre-existing vacancies in symmetrically non-equivalent sites, and at interstitial positions. At high pressure, most structural changes affect the MgO$_{6}$ units and the enthalpies of point defects increase, with those involving Mg and Si vacancies increasing more than those involving O sites. At 15 GPa Si vacancies and Mg interstitials have become the predominant intrinsic defects. We use these calculated enthalpies to estimate the total uptake of noble gases into the bulk crystal as a function of temperature and pressure both in the presence and absence of other heterovalent trace elements. For He and Ne our calculated solubilities point to atoms occupying mainly interstitial sites in agreement with previous experimental work. In contrast, Ar most likely substitutes for Mg due to its larger size and the deformation it causes within the crystal. Incorporation energies, as well as atomic distances suggest that the incorporation mainly depend on the size mismatch between host and guest atoms. Polarization effects arising from the polarizability of the noble gas atom or the presence of charged defects are minimal and do not contribute significantly to the uptake. Finally, the discrepancies between our results and recent experiments suggest that there are other incorporation mechanisms such as adsorption at internal and external interfaces, voids and grain boundaries which must play a major role in noble gas storage and solubility.

## Introduction

Noble gas behaviour is extremely important as the extent of noble gas incorporation into mantle minerals controls models for degassing of the Earth^1–9^, the nature of mantle convection^10–14^ and underpins several radiometric dating methods^15–17^. Most geochemical models for the behaviour of noble gases have explicitly assumed complete incompatibility in minerals so that, during any process involving solid and liquid, the noble gas partitions exclusively into the melt, i.e the partition coefficient *D*, the ratio of the concentrations of the trace element (here the noble gas) in the solid and liquid phases, is zero^9,18–25^.

The literature on noble gas partitioning is somewhat scattered and suggests a range of different values of *D*^24^. Early experimental data on the noble gases indicated that noble gases were not as incompatible as suggested and that some remain in the solid mantle during melt extraction^18,24,26,27^. Results of Broadhurst et al.^28^ support moderate partition coefficients ($0.01<D<47$) between olivine/melt and diopside/melt; solubilities in solid minerals increased with increasing noble gas atomic number and typical solubilities were surprisingly high. However, subsequent work of Brooker et al.^18^ and Heber et al.^23^ concluded that noble gases were more incompatible but not to the extent suggested by current geochemical models^9,29^. In addition, all earth-evolution models have assumed that all noble gases are equally incompatible in all crystal-melt processes, even though there is also evidence of some variation from one noble gas to another^23,30,31^. Interestingly, a separate investigation on the diffusion of helium in MgO and its uptake of argon and helium by solid minerals such as quartz, corundum, enstatite, and olivine unexpectedly revealed significant concentrations of these trace elements (approximately 1000 ppm)^22,32^. If so, there are major implications for the degassing processes of Earth and other terrestrial planets^21^.

Key to the possible incorporation of trace elements including noble gases in bulk minerals are the energies of point defects in these systems such as vacancies and interstitials. Such defects are also of considerable interest for understanding processes such as diffusion, creep, electric conductivity and the transmission and attenuation of seismic signals and the evolution of, for example, the mantle and core. Vacant sites generated extrinsically or intrinsically can incorporate trace elements and their presence is a possible rationalization of measured concentrations. Cation and anion vacancies in minerals have been subject to extensive series of theoretical^33–37^ and experimental studies.

The importance of the storage of trace elements in the mantle has been much discussed and extensively studied for olivine ((Mg,Fe)$_{2}$SiO$_{4}$) owing to its abundance in the upper mantle and the upper crust^37,38^. In this work, ab initio calculations are used to gain insight into point defect formation and incorporation of He, Ne and Ar in forsterite, both in the absence and presence of extrinsic vacancies present due to heterovalent impurities. In “Results”, full details of computational methods used are given, results and discussion for defect stability and noble gases uptake are presented in “Discussion”.

## Results

### Defect enthalpy

**Table 1:** Calculated lattice parameters and bond distances for a 2 $\times$ 1 $\times$ 2 supercell of pure forsterite (Å) at different pressures. $^{\dagger }$ Experimental values for 0 GPa from Schwab and Küstner (1977). Also listed are the fractional coordinates of the atoms in the asymmetric unit of forsterite together with those of the interstitial position used for the noble gas atoms.

Parameter	Pressure (GPa)			
	0	5	10	15
*a*	4.80	4.75	4.71	4.69
*b*	10.30	10.14	9.99	9.87
*c*	6.04	5.96	5.89	5.83
Si-O1	1.63	1.62	1.61	1.60
Si-O2	1.67	1.66	1.65	1.64
Si-O3	1.66	1.65	1.64	1.63
M1-O1	2.11	2.08	2.05	2.03
M1-O2	2.09	2.07	2.05	2.03
M1-O3	2.15	2.12	2.10	2.08
M2-O1	2.20	2.15	2.12	2.09
M2-O2	2.07	2.04	2.01	1.99
M2-O3	2.09	2.06	2.03	2.00
Asymmetric unit coordinates				
	x	y	z	
M1	0.0000	0.0000	0.0000	
M2	0.9896	0.2776	0.2500	
O1	0.7667	0.0918	0.2500	
O2	0.2202	0.4477	0.2500	
O3	0.2781	0.1633	0.0337	
Si	0.4226	0.0945	0.2500	
NG	0.3277	0.9052	0.1096	

**Figure 1:**
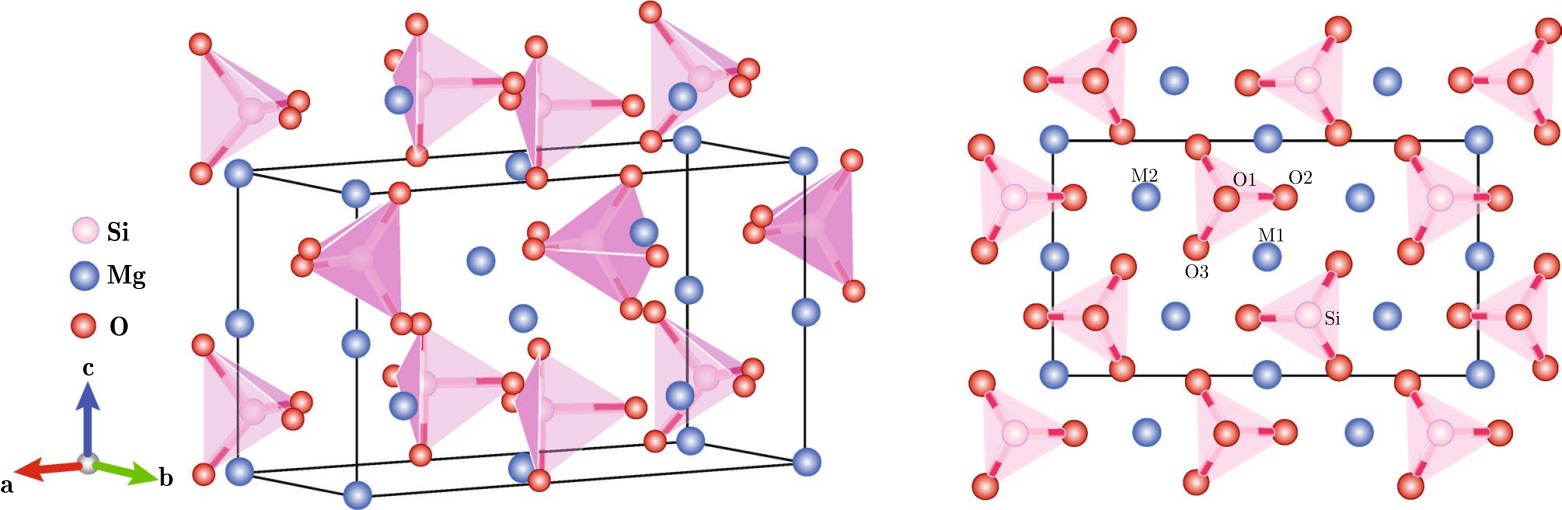
Atomic structure of forsterite, showing the different non-equivalent Mg atomic sites M1 and M2, the Si site and the three different O sites, O1, O2 and O3.

We first present results for the changes in the structural parameters of forsterite at zero temperature over a range of pressures from 0 to 15 GPa. Figure 1 shows the unit cell of forsterite showing the non-equivalent oxygen sites (O1, O2 and O3), the two non-equivalent metal sites (M1 and M2) and the Si position. The optimized cell parameters and a set of representative interatomic distances at different pressures are presented in Table 1. Agreement between theory and experiment is good. Lattice parameters differ by less than 1$\%$ from the experimental values reported by Schwab and Kustner et al.^39^, Finkelstein et al.^40^ and Bouibes and Zaoui^41^ at atmospheric pressure. Our calculations show that the SiO$_{4}$ tetrahedra compress less than the MgO$_{6}$ polyhedra, consistent with the greater stiffness of the Si–O bond. It is known that forsterite shows anisotropic behaviour under compression^42,43^, where the *a*-direction is the least compressible and *b* the most. Our calculations are in agreement with these results.

We investigate the energetics of defect formation by computing the defect enthalpy which we define by:

1 $$\begin{aligned} {H}_{{\textrm{def}}}={H}(\textrm{M}|\textrm{Mg}_{2}\textrm{SiO}_{4})- H (\textrm{Mg}_{2}\textrm{SiO}_{4})-{H}(\textrm{M}) \end{aligned}$$

where $H\mathrm {(M|Mg_{2}SiO_{4})}$ is the enthalpy of the system containing a defect of type M, $H\mathrm {(Mg_{2}SiO_{4})}$ the enthalpy of the perfect solid and $H( M )$ the enthalpy of the isolated noble gas atom.

Supercell calculations for charged systems must always include a compensating homogeneous background charge in order to neutralize the net charge of the supercell. The unphysical electrostatic interaction of the defect with its periodic images and the constant background makes a spurious contribution to the calculated energy of the system^44–46^. For cubic systems, a straightforward suggested correction involves an equation that incorporates the supercell lattice parameter and the Madelung constant^45,46^. In the case of systems with lower symmetry, like forsterite, the correction can be approximated by calculating the electrostatic energy of the charged array^36^ as follows:

2 $$\begin{aligned} E_{c}=\frac{\beta q^2}{\varepsilon _0} \end{aligned}$$

**Table 2:** Summary of total enthalpies (eV) for the perfect (undefective) 112 atom supercell and defect enthalpies, as defined in Eq. (1) for the six possible types of ionic vacancies at different pressures (GPa). The values of the enthalpies both before and after (italics) the Makov–Payne correction for charged supercells are listed.

System	Pressure (GPa)			
	0	5	10	15
Super-cell	$-93521.45$	$-93520.72$	$-93518.88$	$-93516.26$
Mg-M1	1724.68	1724.83	1724.94	1725.06
	$1725.90$	$1726.06$	$1726.19$	$1726.32$
Mg-M2	1725.49	1725.70	1724.94	1726.13
	$1726.70$	$1726.91$	$1726.18$	$1727.39$
O-1	555.30	556.74	556.73	556.45
	$556.53$	$557.98$	$557.99$	$557.73$
O-2	556.66	555.36	555.38	555.08
	$557.89$	$556.61$	$556.65$	$556.36$
O-3	554.47	554.49	554.40	554.06
	$555.71$	$555.74$	$555.66$	$555.34$
Si	194.63	194.98	195.47	195.90
	$199.41$	$199.84$	$200.41$	$200.89$

where *q* is the defect net charge, $\epsilon _0$ the dielectric constant and the factor $\beta$ depends on the cell parameters and varies from 2.4 to 2.6 over the range of studied pressures. Defect enthalpies at various pressures are given in Table 2. We list the values of the final enthalpy both before and after the application of the Makov–Payne correction. For the cell size we use this correction is appreciable, always increasing the defect energies, and cannot be neglected.

**Table 3:** Defect reactions in forsterite and the corresponding defect enthalpies (eV) in the static limit at different pressures, using the Kröger–Vink notation. Results of Broadhurst et al. (1990) $^{\dagger }$ ^38^ and Wright et al. (1991)$^{\dagger \dagger }$ ^37^ are given for comparison.

Reaction	Pressure (GPa)			
	0	5	10	15
Mg$_{\text {Mg}}+$V$$_{\text {i}}\longrightarrow \ $$Mg$_{\text {i}}^{^{\bullet \bullet }}+$V$_{\text {Mg}}^{^{\prime \prime }}$	7.67	10.08	11.56	13.02
Si$_{\text {Si}}+$V$$_{\text {i}}\longrightarrow \ $$Si$_{\text {i}}^{^{\bullet \bullet \bullet \bullet }}+$V$_{\text {Si}}^{^{\prime \prime }}$	26.05	30.79	34.20	36.38
O$_{\text {O}}+$V$$_{\text {i}}\ \longrightarrow \ $$O$_{\text {i}}^{^{\prime \prime }}+$V$_{\text {O}}^{^{\bullet \bullet }}$	9.40	9.50	9.56	9.59
Mg$_{\text {2}}$SiO$$_{\text {4}}\ \longrightarrow \ $$2V$_{\text {Mg}}^{^{\prime \prime }} +$V$_{\text {Si}}^{^{^{\prime \prime \prime \prime }}} +$4V$_{\text {O}}^{^{\bullet \bullet }}$	36.46	38.11	40.33	43.10
	30.25$^{\dagger }$, 37.4$^{\dagger \dagger }$			
Mg$_{\text {Mg}}+$O$$_{\text {O}}\ \longrightarrow \ $$V$$_{\text {Mg}}^{^{\prime \prime }}+\ $$V$_{\text {O}}^{^{\bullet \bullet }}$	7.31	9.32	11.74	14.64
	8.40 $^{\dagger \dagger }$			

At zero pressure a vacancy at O3 is only 1.0 eV lower in enthalpy than at O1 but 2.1 eV lower than at O2. A vacancy at a M1 position is 0.8 eV lower than a vacancy at M2. The enthalpies of vacancies at M1 and M2 show little change with pressure, with increases of $\approx$ 0.42 eV and $\approx$ 0.70 eV from 0 to 15 GPa for M1 and M2 respectively. In contrast, oxygen vacancies at O2 and O3 decrease in energy by − 1.5 eV and 0.4 eV respectively. These differences suggest that most of the structural changes that occur with pressure affect the M1 and M2 sites while the SiO$_4$ unit resists compression. We can use the vacancy enthalpies given in Table 2 to define several types of Schottky and Frenkel defect pairs as shown in Table 3 where we use Kröger–Vink notation^47^. The results for the Schottky defects at zero pressure are in good agreement with previous work^37,43^. For the Schottky defects, the pair V$_{\text {Mg}}^{\prime \prime }$+V$_{\text {O}}^{^{\bullet \bullet }}$ is lowest in energy with a formation enthalpy of 7.31 eV at 0 GPa. This energy increases to 14.64 eV at 15 GPa. Overall, our calculations show that defects created involving the formation of Mg and Si vacancies tend to be energetically more expensive as pressure increases than those related to O sites. All defect energies shown in Table 3 increase with increasing pressure; this is more clearly seen for the defects involving M1 or M2 sites and presumably related to the bond compressibility since the M1–O bond is more compressible than any of the Si–O bonds.

The difference in defect enthalpy between vacancies of the same type of atom located at non-equivalent sites determines the relative number of defects at different sites. This ratio is proportional to $e^{-\Delta H/k_{B}T}$, where $\Delta H$ is the enthalpy difference between the two non-equivalent vacancy sites, $k_{B}$ the Boltzmann constant and *T* the temperature. In general, the total uptake of trace elements depends on the mechanism of incorporation which in turn is a function of the defect energies^48–51^. Any estimate of the total concentration of noble gas in the solid also needs to take into account the concentration of vacancies and interstitial sites available in the crystal as a function of temperature and pressure.

According to defect theory^52^, at equilibrium, the concentrations [*A*], [*B*] and [*C*] in the defect reaction *A* + *B* $\rightarrow$ *C*, are related by:

3 $$\begin{aligned} \frac{\left[ C\right] }{\left[ A\right] \left[ B\right] }=K_{eq}=\exp \left[ -\frac{\Delta G\left( T\right) }{k_BT} \right] , \end{aligned}$$

where $\Delta G=G(C)-G(A)-G(B)$ is the free energy of the reaction, $k_{B}$ the Boltzmann constant and *T* the temperature. We work in the dilute limit since defect concentrations are small and activity coefficients close to one^29,53^.

Pinilla et al.^50^ showed that defect enthalpies change only slighly with temperature in forsterite, consistent with the general thermodynamic arguments of Harding et al.^54^ and Taylor et al.^55^. These show that a somewhat surprising accurate approximation for the defect enthalpy, $h_P(T)$, at any temperature is the internal energy change at 0 K, $u_v(0)$ . In contrast, the pressure variation shown in Table 3 over the pressure 0–15 GPa is much larger. Thus we have neglected the temperature variations of the defect enthalpies. Point defect entropies are neglected; their magnitudes^54,55^ are sufficiently small such that the differences between $g_P(T)$ and $h_P(T)$ are likely not to exceed an eV. In addition, Pinilla et al.^50^ previously compared noble gas solution energies calculated from a full free energy minimisation with those determined making this approximation. The differences are small. All conclusions in the present paper are unchanged by this approximation.

The equations in Table 3 need to be supplemented by another relation imposing charge neutrality:

4 $$\begin{aligned} \left[ \text {V}_{\text {Mg}}^{\prime \prime }\right] +2\left[ \text {V}_{\text {Si}}^{\prime \prime \prime \prime }\right] +\left[ \text {O}_{\text {I}}^{\prime \prime }\right] -\left[ \text {V}_{\text {O}}^{^{\bullet \bullet }}\right] -\left[ \text {Mg}_{\text {i}}^{^{\bullet \bullet} }\right] -2\left[ \text {Si}_{\text {i}}^{^{\bullet \bullet \bullet \bullet }}\right] =0, \end{aligned}$$

so providing a closed set of six equations containing the six unknown defect concentrations: $\left[ \text {V}_{\text {Mg}}^{\prime \prime }\right]$, $\left[ \text {V}_{\text {Si}}^{\prime \prime \prime \prime }\right]$ , $\left[ \text {O}_{\text {I} }^{\prime \prime }\right]$, $\left[ \text {V}_{\text {O}}^{^{\bullet \bullet }} \right]$, $\left[ \text {Mg}_{\text {i}}^{^{\bullet \bullet }}\right]$ and $\left[ \text {Si}_{\text {i}}^{^{\bullet \bullet \bullet \bullet} }\right]$.

**Figure 2:**
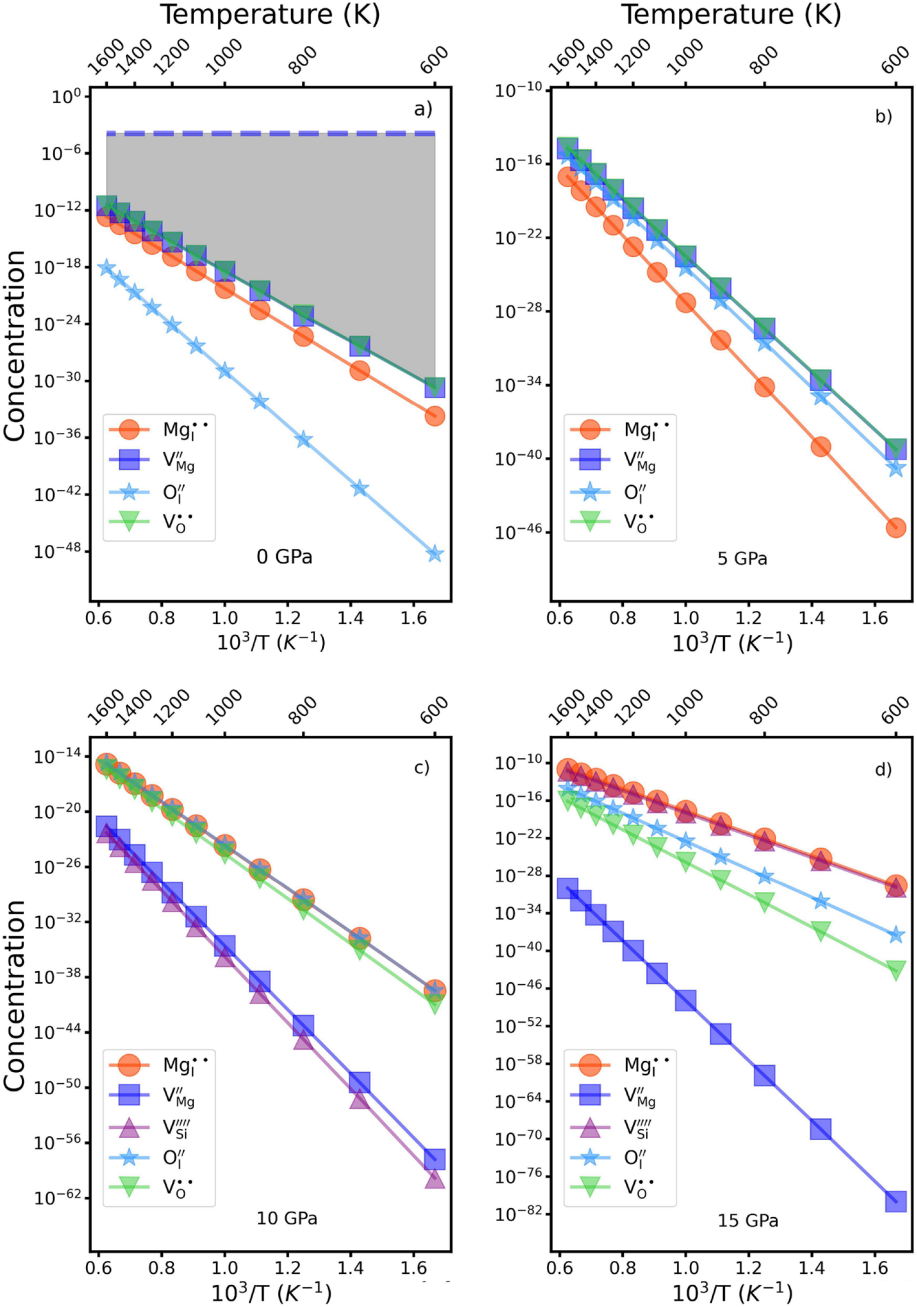
Full lines show the estimated concentrations (in mole fraction) for intrinsic defects in a pure sample of forsterite at different pressures and as a function of temperature. In (**a**) the dashed line shows the concentration of Mg vacancies for crystals containing 200 ppm (2.7 $\times$ 10$^4$ mole fraction) of Cr$^{3+}$ as described in the text. The grey shaded area thus shows the possible values for the concentration of magnesium vacancies. The lower limit (full line, squares) represents the scenario in which only intrinsic defects are present and the upper limit (dashed line) that when extrinsic defects are also present.

Solving this system of equations using the energy values from Table 3 yields the temperature variation of the concentrations of the different defects as shown in Fig. 2 for the studied pressures. Mg and O vacancies are present in the highest concentrations varying from 10$^{-12}$ to 10$^{-5}$ mol fraction (i.e. per formula unit of forsterite) over the temperature range 700–1600 K at 0 GPa. Si vacancies are present at much lower concentrations by orders of magnitude, which reflects the high Si–O bond energy. Thus the principal intrinsic defects change with increasing pressure and at 15 GPa silicon vacancies and magnesium interstitials are the most important. This again reflects the structural changes seen earlier.

### Defect concentration and uptake of noble gases in forsterite

The total solubility of a noble gas is closely linked to the number of sites present in the solid which can act as traps. Using statistical mechanics within the grand canonical ensemble, the total solubility of noble gas atoms in a solid can be written as a function of the different defect concentrations following Lidiard^56^:

5 $$\begin{aligned} C_{T}=\sum _{i=1}^{n}C_{i}(P,T)=\frac{Ph^{3}}{(2\pi m)^{3/2}(k_{B}T)^{5/2}} \sum _{i=1}^{n}[A_{i}]e^{-g_{i}/k_{B}T} \end{aligned}$$

**Table 4:** Calculated enthalpies of insertion of a noble gas atom (He, Ne, Ar) at symmetrically non-equivalent lattice sites and at the interstitial site.

$\Delta H_{\text{ def }}\left( \text {eV}\right)$	Pressure (GPa)			
	0	5	10	15
He				
M1	0.56	0.68	0.70	0.77
M2	0.47	0.60	0.93	0.97
O1	1.84	1.91	2.25	2.73
O2	1.10	1.20	1.75	2.21
O3	1.20	1.20	1.37	1.67
Si	0.64	0.69	0.73	0.95
Interst.	1.00	1.09	1.14	1.33
Ne				
M1	1.64	1.94	2.09	2.31
M2	1.39	1.69	2.03	2.19
O1	2.27	3.23	3.40	5.10
O2	1.39	2.44	3.32	4.89
O3	1.44	2.68	3.57	5.29
Si	1.95	2.20	2.26	2.62
Interst.	2.45	3.19	3.80	4.28
Ar				
M1	3.59	4.37	4.73	5.28
M2	3.22	4.06	4.72	5.19
O1	3.43	3.87	4.27	5.14
O2	2.57	3.55	3.96	4.20
O3	2.51	3.23	3.91	4.29
Si	2.49	2.66	2.97	3.19
Interst.	5.72	5.90	6.26	6.78

where [$A_{i}$] is the mole fraction concentration of sites (traps) of type *i* per formula unit of forsterite, *P* is the partial pressure of the gas and *T* the temperature. The set of the [$A_{i}$] includes incorporation at different vacancies and interstitial positions, and also substitutions at Mg, O and Si sites. Making the same approximation as above, we use enthalpy differences instead of free energies^57^ ($g_i \approx h_{i}$) to calculate the total concentrations from Eq. (5). $h_{i}$ is the work done at constant pressure and temperature to bring a gas atom from a state of rest at infinity and place it in a particular site (trap) *i* (see Table 4).

**Figure 3:**
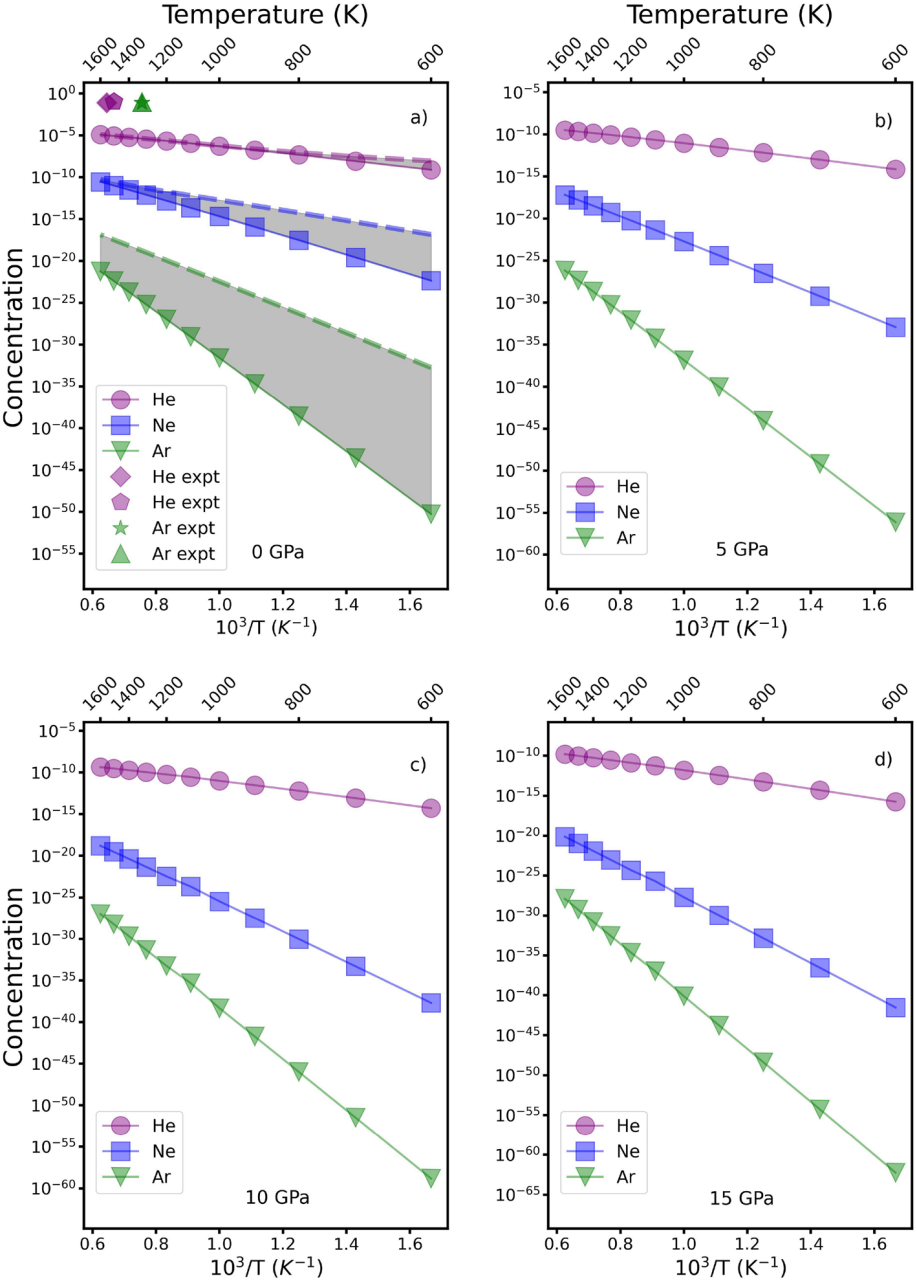
Estimated concentrations ( in mole fraction) of He (circles, full purple line), Ne (squares, full blue line) and Ar (triangles, full green line) in forsterite in the absence of extrinsic defects as a function of temperature for different pressures. The dashed lines in panel (**a**) show the corresponding concentration for crystals containing 200 ppm (2.7 $\times$ 10$^4$ mole fraction) of Cr$^{3+}$. For each gas, the gray shaded area thus shows possible values of noble gas uptake; the lower limit (full lines) is the scenario where only intrinsic defects are present and the upper limit (dashed lines) that when extrinsic defects are also present. Experimental values for He from Parman et al.^20^ (purple pentagon), Delon et al.^58^ (purple diamond) and for Ar from Delon et al.^59^ (green star) and Baxter et al.^60^ (green triangle) are given for comparison.

The enthalpy values $h_{i}$ in Table 4, which we have used in Eq. (5), were obtained from the GGA ab-initio calculations as described earlier. Figure 3 shows the calculated concentrations $C_{T}$ of trapped He, Ne and Ar in forsterite as a function of temperature. The incorporation increases with increasing temperature and decreases at high pressures. As expected, the total He concentrations are larger than those obtained for Ne and Ar related to the size difference between these atoms. We compare the obtained concentrations with values for He and Ar taken or estimated from Parman et al.^58^, Delon et al.^59^ and Watson et al.^22^. For He our values are in fair agreement with the experimental work. However, a much larger difference is seen for Ar where the calculated uptake due to single point defects and the obtained experimental values differ by more than 10 orders of magnitude.

## Discussion

We have examined several incorporation mechanisms including interstitial and substitution incorporation, and see that the incorporation of noble gases depends on their atomic size, the pressure and the temperature. The effects of pressure and temperature are less pronounced than atomic size. For He, even though the incorporation at pre-existent M1 and M2 vacancy sites is the most energetically favorable, the number of such sites is much smaller than the number of interstitial sites so incorporation at interstitial sites predominates. For Ne and Ar this analysis is more complicated. At low pressure O2 and O3 vacancies together with Si vacancies are all relevant for the incorporation of Ne and Ar. At the highest studied pressure, incorporation at Si vacancies are the most stable incorporation mechanism. As we have seen Si vacancies start to become more accessible when the pressure increases as shown in Fig. 2.

Often, experimental studies of the storage of noble gases in olivine at upper mantle conditions include the estimation of the segregation factor $s=C^{GB}/C^{LAT}$ that relates the concentration of noble gas present at grain boundaries $C^{GB}$ and the crystal lattice $C^{LAT}$ at thermodynamic equilibrium^61,62^. This quantity depends on the interface area available and thus the mantle grain size ($\approx$ 10$^{-2}$ to 10$^{-4}$ m^63^). In Fig. 3 we have displayed the estimated values for the uptake of He using the data reported by Parman et al.^20^, Delon et al.^58^ and Delon et al.^61^, where the total He solubility ranges from 0.78 to 0.99 mole fraction. Note that these experiments report the partitioning coefficient of noble gases between solid silicate and melt. We have estimated the corresponding solid solubility assuming that the melt and the noble gas form an ideal solution. This approximation has been discussed extensively in the literature^64,65^; the assumption of ideality relies on low solubilities, elevated temperatures, the structure of the melt and the absence of interactions in the flexible liquid other than the weak dispersion forces.

Our calculated solubility of He in Fig. 3 agrees well with that estimated from experiment and shows that for a light element such as He, lattice storage at interstitial sites and prexisting vacancies play an important role in the uptake of He. It is however, important to mention, as also stressed earlier by Pinilla et al.^50^ and Delon et al.^58^, that grain boundaries can also play a role and that for small grain sizes they can be responsible of up to 75% of the total uptake and cannot be neglected when modelling mantle dynamics processes involving noble gases. In contrast, the experimental estimation of Ar uptake in olvine is often more challenging due to the slow diffusion of Ar within the lattice and thus values of *s* cannot be readily obtained. Only one study has attempted to determine *s* for Ar at mantle minerals^60^, with values lying between 1.45 $\times$ 10$^5$ and 4.17 $\times$ 10$^4$ in polycrystalline diopside. Another method of estimating *s* for Ar has involved the estimation of the partition coefficient between olivine and a silicate melt. However, these values are currently poorly constrained and vary over several orders of magnitude^18,22,23,28,32^. Recent work^59^ has suggested that since most of these data has been obtained from samples whose surfaces were previously polished before analysis, atmospheric contamination may have increased the reported partition coeffcient ($K^{ol-melt}$) values^50,59^. Indeed, values for Ar solubility in olivines have been reported of the order of $\approx$ 1000 ppm^22,32^ which are so much higher than estimates of bulk solubility so we conclude that Ar uptake occurs mainly at interfaces. Figure 3 compares the estimated values reported by Delon et al.^59^ and Baxter et al.^60^ with our estimated values. It is clear that uptake in the bulk is several orders of magnitude lower than reported experimentally, suggesting that the mechanisms of incorporation such as grain boundaries must be relevant for the uptake of such a large atom and that lattice uptake is negligible^50^. Our value is also much lower than the estimated solubility of Ar estimated by Karato^24^ using the partitioning results of Heber et al.^23^ and Brooker et al.^18^. Karato’s estimate for Ar is within a factor of five of his similar estimate for He, with which our calculated value in this paper is in good agreement. Finally, our findings indicate that the uptake of noble gases should decrease as pressure increases. However, it also increases with temperature, suggesting that the $e^{-g/k_BT}$ term dominates, even though the free energy of the gas is also lower since the entropy of the gas increases.

In addition to iron, natural olivines contain a wide range of impurities present at various concentration levels, which can lead to a substantial population of extrinsic defects if these trace elements are heterovalent, i.e. have a different oxidation state from the ion for which they substitute^52^. Among the significant elements that can be incorporated in natural samples, such as San Carlos olivine, often used as a reference for magmatic processes, are Fe$^{3+}$ and Cr$^{3+}$, which preferentially occupy Mg sites^51^. Cr$^{3+}$ appears to be the dominant trivalent species in olivine when Fe$^{3+}$ is disregarded. To date, accurate determination of the total amount of Fe$^{3+}$ has proven challenging and typically yields results below Mössbauer Spectroscopy resolution (approximately 100 ppm), whereas Cr$^{3+}$ concentrations can reach values of up to 200 ppm in mantle olivine and 1000 ppm in magmatic olivine^66,67^. Consequently we have also assessed the uptake of noble gases, considering Cr$^{3+}$ as a substitution impurity occupying Mg sites. To a good approximation, at least at lower pressures, the presence of two Cr$^{3+}$ species at Mg sites generates a single $\left[ \text {V}_{\text {Mg}}^{\prime \prime }\right]$ to maintain charge neutrality within the mineral. In this case Eq. (4) becomes:

6 $$\begin{aligned} 2\left[ \text {V}_{\text {Mg}}^{\prime \prime }\right] +4\left[ \text {V}_{\text {Si}}^{\prime \prime \prime \prime }\right] +2\left[ \text {O}_{\text {i}}^{\prime \prime }\right] - \left[ \text {Cr}_{\text {Mg}}^{\hspace{1.0mm}\bullet }\right] - 2\left[ \text {V}_{\text {O}}^{\bullet \bullet }\right] -2 \left[ \text {Mg}_{\hspace{1.0mm}\text {i}}^{\bullet \bullet }\right] - 4\left[ \text {Si}_{\hspace{2.0mm}\text {i}}^{\bullet \bullet \bullet \bullet }\right] = 0. \end{aligned}$$

Solving this modified system of equations, taking $\left[ \text {Cr}_{\text {Mg}}^{\hspace{1.0mm}\bullet }\right]$ equal to 200 ppm (2.7 $\times$ 10$^{-4}$ mole fraction), the predominant defect are to Mg vacancies with mole fractions approaching 10$^{-4}$, as depicted in Fig. 2. This has important implications due to the number of sites available for the incorporation of noble gases within the olivine structure. Taking this into consideration, the estimated noble gas uptakes fall within the grey shaded regions illustrated in Fig. 3. The lower limit represents the scenario where only purely intrinsic defects are considered, while the upper limit (dashed line) accounts for the presence of extrinsic defects. We stress that the total concentration of noble gases incorporated at the mineral remains significantly lower than that reported in previous experimental studies, especially for Ar^22,32^.

**Table 5:** Average distances to nearest neighbours in Å for noble gas atoms occupying interstitial positions, M2 and Si sites in forsterite at 0 GPa. Only values from configurations with the lowest enthalpy are shown. For comparison, Lennard–Jones potential parameters $\sigma$ for noble-gas/oxygen interactions are approximately 2.42 Å, 2.75 Å and 3.06 Å for He–O^50^, Ne-O^49^ and Ar–O^49^ respectively.

He			
Distance	Interst.	M2	Si
He–Mg	2.40	3.37	3.11
He–O	2.22	2.40	2.41
He–Si	2.28	3.08	4.20
Ne			
Distance	Interst.	M2	Si
Ne–Mg	2.46	3.42	3.14
Ne–O	2.30	2.46	2.48
Ne–Si	3.72	3.17	4.27
Ar			
Distance	Interst.	M2	Si
Ar–Mg	2.52	3.47	3.27
Ar–O	2.30	2.59	2.95
Ar–Si	3.62	3.30	3.12

Thomas et al.^32^ have suggested that polarization is the driving mechanism for the incorporation of noble gas atoms in the lattice and that this might explain the large concentrations seen by these authors. In this context we have examined the interatomic separations between each noble gas and its nearest neighbours as shown in Table 5. We have compared the shortest of these distances, in each case that between the noble gas and oxygen, with the corresponding values of the Lennard–Jones size parameter^49^, $\sigma$, below which the noble-gas-oxygen potential is repulsive. In every case the interatomic distances are much less than $\sigma$, consistent with the high positive energies of noble gas incorporation, indicating strong repulsion between the noble gas and its nearest oxygen neighbours and a negligible role played by attractive dispersion and polarization. Note also that the polarizability of the oxide anion^68^ is approximately three times that of Mg$^{2+}$ and nine times that of Si$^{4+}$ so noble-gas-cation dispersion interactions will be much smaller. Atomic size dominates; electronic polarization as suggested by Thomas et al. is not important.

Previous work has found low activation energy barriers for the diffusion of noble gas species into the crystal^50,69,70^. The size of the noble gas guest atoms are such that the neighbouring lattice is greatly deformed, the calculated energy landscapes are flat and, as described by Pinilla et al.^50^, migration from one local minimum to the next does not involve overcoming high energy barriers. This leads to high noble gas mobility and therefore to likely segregation of the noble gas to the surface over geologically relevant timescales.

## Conclusions

This work brings new insights into the defect chemistry of the common mantle mineral olivine. There are dramatic changes in the nature of the predominant intrinsic defects with pressure. We then considered the incorporation of noble gases in olivine and especially their storage under upper mantle conditions. In particular, we concentrated on understanding the effects that intrinsic point defects and available interstitial sites can have on the incorporation of He, Ne and Ar. Our work allows us to estimate the maximum noble gas concentrations that can be incorporated in the mineral, if only point defects are considered as a function of temperature and pressure. We have seen that pressure and noble gas atomic size are factors that greatly affect the incorporation of noble gases. Our work shows that the availability of interstitial sites, together with M site vacancies, can be responsible for most of the uptake of small size noble gas atoms such as He and Ne. Our results suggest that, for Ar uptake, Si vacancies could also be relevant, especially at high pressures. The total concentrations we estimate are very low, of the order of 10$^{-3}$ mole fraction for He and 10$^{-8}$ mole fraction for Ar at 0 GPa and 1400 K. The results for He are in good agreement with estimates from experimental data of Parman et al.^20^ and Delon et al.^58^. However, large discrepancies are found between our results and experiments of Thomas et al.^32^ even allowing for reasonable concentrations of likely heterovalent trace elements which increase the concentration of vacancies. Our ab-initio results show that the energy of incorporation are largely driven by size mismatch between host and guest atom and that polarization effects arising from the presence of a charged vacancy are minimal and will not contribute significantly to the uptake. While our study focuses on the incorporation at single point defects, it is evident that extended defects like concentrated vacancy clusters and grain boundaries, rather than point defects, play a crucial role in the uptake of noble gases.

## Methods

Computations have been performed using density functional theory (DFT) methods^71,72^, as implemented in the Quantum Espresso package^73,74^, within the Generalised Gradient Approximation (GGA) with the Perdew–Burke–Ernzerhof (PBE)^75,76^ approximation. We have used ultrasoft pseudopotentials for Mg, O, Si, He, Ne and Ar.

The irreducible Brillouin zone has been sampled with a set of 4 Monkhorst and Pack special points^77^ with solid cohesive energies differing by less than 0.001 eV from calculations using a finer grid of 18 special *k*-points. Supercells containing a total of 111, 112 or 113 atoms were used for defect calculations, depending on the number of defects. Calculations run using a larger supercell of 224 ions showed that defect energies were typically converged to 0.001 eV.

Optimization of atomic positions and where specified also unit-cell parameters were carried out using GGA. The defect energy is defined as the difference of energy between a defect-free supercell and one containing a single defect of a given type^55^. Three different types of single charged vacancies are defined: Mg$^{2+}$, Si$^{4+}$ and O$^{2-}$. Vacancies and interstitial are defined by removing or adding atomic cores and the appropriate number of valence electrons. Electrically neutral combinations of these vacancies form Schottky defects. A charged background with the appropriate corrections is used to neutralize the net charged supercell and thus calculate contributions from long-range interactions^46^.

We calculate enthalpies for the incorporation of noble gas atoms at interstitial sites and at intrinsic vacancies, and enthalpies of substitution at occupied lattice sites. For interstitials we tried different locations and found that the lowest energy position lies between the O2, O3 and M2 atoms. The calculated basis atom coordinates for forsterite and that of the interstitial position are collected together in Table 1. Nevertheless, as shown in previous work on He defects in $\alpha$-Fe^50,78,79^ and zircon^80^, long-range dispersive attraction is not expected to make a large contribution to the defect enthalpy since the interatomic distances are such that repulsion between the electron clouds of the trace element and the host atoms dominates.

## Data Availability

The dataset and relaxed structures used in the current study are available from the corresponding author on request.
